# Circulating miR-320a Acts as a Tumor Suppressor and Prognostic Factor in Non-small Cell Lung Cancer

**DOI:** 10.3389/fonc.2021.645475

**Published:** 2021-03-23

**Authors:** Akanksha Khandelwal, Uttam Sharma, Tushar Singh Barwal, Rajeev Kumar Seam, Manish Gupta, Manjit Kaur Rana, Karen M. Vasquez, Aklank Jain

**Affiliations:** ^1^Department of Biochemistry and Microbial Sciences, Central University of Punjab, Bathinda, India; ^2^Department of Zoology, Central University of Punjab, Bathinda, India; ^3^Department of Radiation Oncology, Indira Gandhi Medical College, Shimla, India; ^4^Lab Medicine, Department of Pathology, All India Institute of Medical Sciences, Bathinda, India; ^5^Division of Pharmacology and Toxicology, College of Pharmacy, Dell Pediatric Research Institute, The University of Texas at Austin, Austin, TX, United States

**Keywords:** miR-320a, NSCLC, liquid biopsy, cancer, prognosis

## Abstract

Dysregulated expression profiles of microRNAs (miRNAs) have been observed in several types of cancer, including non-small cell lung cancer (NSCLC); however, the diagnostic and prognostic potential of circulating miRNAs in NSCLC remains largely undefined. Here we found that circulating miR-320a was significantly down-regulated (~5.87-fold; *p* < 0.0001) in NSCLC patients (*n* = 80) compared to matched control plasma samples from healthy subjects (*n* = 80). Kaplan-Meier survival analysis revealed that NSCLC patients with lower levels of circulating miR-320a had overall poorer prognosis and survival rates compared to patients with higher levels (*p* < 0.0001). Moreover, the diagnostic and prognostic potential of miR-320a correlated with clinicopathological characteristics such as tumor size, tumor node metastasis (TNM) stage, and lymph node metastasis. Functionally, depletion of miR-320a in human A549 lung adenocarcinoma cells induced their metastatic potential and reduced apoptosis, which was reversed by exogenous re-expression of miR-320a mimics, indicating that miR-320a has a tumor-suppressive role in NSCLC. These results were further supported by high levels of epithelial-mesenchymal transition (EMT) marker proteins (e.g., Beta-catenin, MMP9, and E-cadherin) in lung cancer cells and tissues *via* immunoblot and immunohistochemistry experiments. Moreover, through bioinformatics and dual-luciferase reporter assays, we demonstrated that *AKT3* was a direct target of miR-320a. In addition, AKT3-associated PI3K/AKT/mTOR protein-signaling pathways were elevated with down-regulated miR-320a levels in NSCLC. These composite data indicate that circulating miR-320a may function as a tumor-suppressor miRNA with potential as a prognostic marker for NSCLC patients.

## Introduction

Despite recent advancements in the diagnosis, prognosis, and treatment of non-small cell lung cancer (NSCLC), the 5-year survival rate is unacceptable at <15% (1). This is due, in part, to the advanced stages at which NSCLC is diagnosed because symptoms in the early stages of NSCLC often coincide with other respiratory diseases, resulting in delayed detection. Bronchoscopy in combination with tissue biopsy is most commonly used for disease confirmation and progression; however, these techniques are invasive and often painful for cancer patients (2–4). Therefore, a blood-based minimally-invasive method for the detection and progression of NSCLC is urgently needed (5, 6).

Small non-coding RNAs, known as microRNAs (miRNAs), disseminate into various body fluids such as plasma, serum, urine, saliva, etc. (7–9). In addition, many miRNAs have been implicated in the pathogenesis of cancer where they can act as oncogenic and/or tumor-suppressive molecules (6–9). Because they disseminate into the circulation, their altered expression in blood samples could be tested prior to the appearance of clinical symptoms in NSCLC patients. Hence, circulating miRNAs may potentially serve as useful biomarkers to screen and manage NSCLC patients (5, 6, 10, 11). Indeed, in a recent study on lung cancer, a plasma-based microRNA signature classifier (MSC) was used to screen high-risk NSCLC individuals, and improved the specificity and reduced the false-positive rate of low-dose computed tomography (LDCT) (12–14). In other reports, plasma miRNAs such as miR-21-5p, miR-141-3p, miR-145-5p, miR-155-5p, and miR-223-3p were found to be up-regulated in tumor node metastasis (TNM) stages I and II of NSCLC patients (13, 15). Similarly, it was reported that up-regulated serum levels of miR-210 were associated with progression-free survival (PFS), while reducing the overall survival (OS) and disease-free survival (DFS) of NSCLC patients (16). Circulating miR-195 expression correlated with clinicopathological characteristics such as lymph node metastasis and advanced clinical stage (17). Further, the diagnostic/prognostic potential of circulating miRNAs was studied where up-regulated plasma levels of miR-16, miR-205, and miR-486 showed a combined specificity of 95% and sensitivity of 80% in NSCLC patients compared to healthy subjects (18).

Several circulating miRNAs have been studied in the context of NSCLC; however, there are currently no specific circulating miRNAs or panels of circulating miRNAs available for the screening and management of lung cancer. Herein, circulating miR-320a was found to be significantly down-regulated in NSCLC samples compared to control samples. Additionally, the down-regulation of circulating miR-320a in NSCLC was associated with TNM stage and poor prognosis of the patients. Mechanistically, *AKT3* was found to be a direct target of miR-320a, and regulated the progression of NSCLC through the PI3K/AKT/mTOR pathway.

## Materials and Methods

### Clinical Sample and Data Collection

The present study was designed according to the Declaration of Helsinki ethical guidelines and was approved by the Ethics Committee of the Regional Cancer Center, Indira Gandhi Medical College, Shimla, Himachal Pradesh, India, and the Central University of Punjab, Bathinda, India. The sample collection was performed according to the predefined inclusion and exclusion criteria. None of the patients received chemotherapy or radiotherapy before the sample collection. Patients were followed for 3 years and the clinical assessment was performed in each patient at the end of the study. Here, NSCLC patients with tumors histopathologically confirmed as either early or metastatic were enrolled, and their clinicopathological features including TNM stage, NSCLC subtype, lymph node status, smoking status, alcoholic status, and age (above 18 years) were also obtained. Similarly, age-matched healthy controls with no smoking or alcohol intake history were enrolled. Individuals (both NSCLC patients and healthy controls) with any disease history, or on medications were excluded from the study. Each enrolled participant was assigned a unique code to maintain their confidentiality and their informed signed consent form was also obtained. Accordingly, peripheral blood plasma samples were obtained from NSCLC patients (*n* = 80) and healthy individuals as controls (*n* = 80) and the samples were stored at −80°C for RNA isolation. Patient follow-up was also performed either by telephone or in person from the hospital outpatient department.

### Circulating miRNA Isolation, cDNA Synthesis, and qRT-PCR Assay

Briefly, 200 μL blood plasma from NSCLC patients and healthy control cohorts was used for circulating miRNA isolation using miRNeasy Serum/Plasma Kit (Qiagen, Inc., Valencia, CA, USA). Isolated circulating miRNAs were quantified by NanoDrop 2000 UV-Vis Spectrophotometer (Thermo Fisher Scientific, Inc., USA). From isolated circulating miRNAs, 100 ng was used as a template for cDNA synthesis in a 20 μL volume using the miScript II RT Kit (Qiagen, Inc., Valencia, CA, USA). After dilution of synthesized cDNA, qRT-PCR was performed to quantify miR-320a expression in NSCLC and healthy control cohorts using the miScript SYBR Green PCR Kit (Qiagen, Inc., Valencia, CA, USA) with a miR-320a gene-specific primer and Ce-miR-39 as a reference control (Qiagen, Inc., Valencia, CA, USA). The qRT-PCR reactions were repeated at least three independent times to avoid any technical variability and the experiments were carried out as mentioned in our previous study (11).

### Overall Survival Using Kaplan-Meier Estimator

Based on the median expression value of miR-320a as cut off value (3.98), NSCLC patients were classified as high expression group (*n* = 40) and low expression group (*n* = 40). We used “1” as the death event while “0” was used if the patient was alive. The survival was estimated using the Kaplan–Meier method, and the survival distributions in association with miR-320a expression were compared between the two groups using the log-rank test (19).

### Cell Culture and Transfection

The human A549 lung adenocarcinoma cell line was given as a generous gift by Dr. Jayant (Central Drug Research Institute, Lucknow, Uttar Pradesh, India). Cell culture was performed as stated in our previous study (11). For transfection experiments, a miR-320a inhibitor (100 nM) (Cat. No.: MIN0000510) and a mimic (100 nM) (Cat. No.: MSY0000510) (Qiagen Inc., Valencia, CA, USA) were used and the procedure was performed as previously described (11).

### Cell Proliferation, Migration, and Invasion Assays

MTT assays were used to study cell proliferation in A549 cells as previously described (11), by transfecting a miR-320a mimic or an inhibitor individually at 1, 5, 10, 20, 50, 100, and 200 nM concentrations. The experiment was repeated at least three independent times. For cell migration and invasion assays, A549 cells were transfected with either a miR-320a mimic (100 nM), an inhibitor (100 nM), or a vehicle control in Opti-MEM reduced-serum media as previously described (11). To determine the effects of miR-320a on cell migration, A549 cells were transfected with 100 nM inhibitor or 100 nM mimic and photographed at 0, 24, 48, and 72 h using 4X magnification of an inverted microscope (Olympus, Hachioji-shi, Tokyo, Japan). For the invasion assay, the cells were observed under 10X magnification by an inverted microscope (Olympus, Hachioji-shi, Tokyo, Japan) 72 h post transfection, and quantified at 560 nm using the BioTek Synergy H1 Hybrid Reader (Winooski, VT, USA).

### Prediction and Validation of miRNA Targets and Pathways

Online available computational target prediction tools *viz*., starBase, TargetScan version 7.2, DIANA-microT version 4, and miRDB, were used to predict direct targets of miR-320a. In addition, KEGG: Kyoto Encyclopedia of Genes and Genomes was utilized to identify potential pathways altered by miR-320a in NSCLC. To validate targeted genes and pathways, cell lysates were prepared from untreated A549 cells and compared to those treated with a miR-320a mimic (100 nM), a miR-320a inhibitor (100 nM), or the vehicle control. Approximately 30 μg of protein extract was used to evaluate the target protein levels using primary antibodies of anti-AKT3 (Cat. No.: 14982), anti-pAKT (pThr308) (Cat. No.: 13038), AKT3 phosphorylated at Thr305, anti-PI3K p110α (Cat. No.: 4249), anti-mTOR (Cat. No.: 2983), anti-phospho-mTOR (pSer2448) (Cat. No.: 5536), anti-Caspase 3 (Cat. No.:9662), anti-p27 (Cat. No.: 3686), anti-Beta-catenin (Cat. No.:8480), anti-MMP9 (Cat. No.: 13667), anti-E-cadherin (Cat. No.: 3195) purchased from Cell Signaling Technologies, Inc., Danvers, Massachusetts, USA, while anti-Cyclin D1 (Cat. No.: C7464), anti-Bcl-2 (Cat. No.: SAB4500003) were purchased from Sigma-Aldrich, Inc., St. Louis, MO, USA. Anti-Beta-actin antibody (Cell Signaling Technologies, Inc., Danvers, Massachusetts, USA; Cat. No.: 4970) was used as a normalization and loading control. The immunoblot assays were performed as previously described (11).

### Immunohistochemistry

Formalin-fixed, paraffin-embedded primary tissue sections were obtained from NSCLC patients. Immunohistochemistry (IHC) was performed at Advanced Cancer Institute, Bathinda, Punjab, India, as previously described (11) and immunostained with primary antibodies against Cyclin D1, Bcl-2, MMP-9, E-cadherin, PI3K, AKT3, phospho-AKT3 (pThr305), mTOR, and phospho-mTOR (pSer2448) using dilutions of 1:50 followed by incubation with Rabbit IgG secondary antibody and photographed after visualizing by Nikon microscope (Japan) in 40X magnification.

### Dual-Luciferase Reporter Assay

The 3′UTR sequence of *AKT3* mRNA was cloned in the vector pMirTarget downstream of the Renilla luciferase gene. All plasmids along with the empty pMirTarget expression vector as a negative control (Cat. No.: PS100062) were purchased from Origene Technologies Inc., Rockville, MD, USA. The plasmid containing the 3′UTR of *AKT3* (NM_001370074) with the predicted binding sites for miR-320a was named “WT-3′UTR AKT3” as wild-type (Cat. No.: CW304801), while the miR-320a binding site was mutated by replacement with other nucleotides in the mutated plasmid, “MUT-3′UTR AKT3” (Cat. No.: CW304802) (**Figure 6B**). These plasmids were used to perform dual-luciferase reporter assays to validate binding of miR-320a on 3′UTR of *AKT3* using the Dual-Luciferase Reporter Assay System (Promega, WI, USA, Cat.No.: E1910) and GloMax 20/20 luminometer (Promega, WI, USA, Cat. No.: E5311) as described previously (11).

### Statistical Analysis

All statistical analyses, such as unpaired and paired student's *t*-test, etc. were performed using GraphPad Prism software version 7. The data are presented in the form of either mean ± standard deviation (SD) or mean ± standard error mean (SEM). Kaplan-Meier method with the log-rank test was used to evaluate NSCLC patient survival between the high and low expression of circulating miR-320a. High and low were defined by designating the median value as the cutoff value (3.98). For all data, *p* < 0.05 was considered as statistically significant.

## Results

### Circulating miR-320a and Its Association With NSCLC

**Figure 1:**
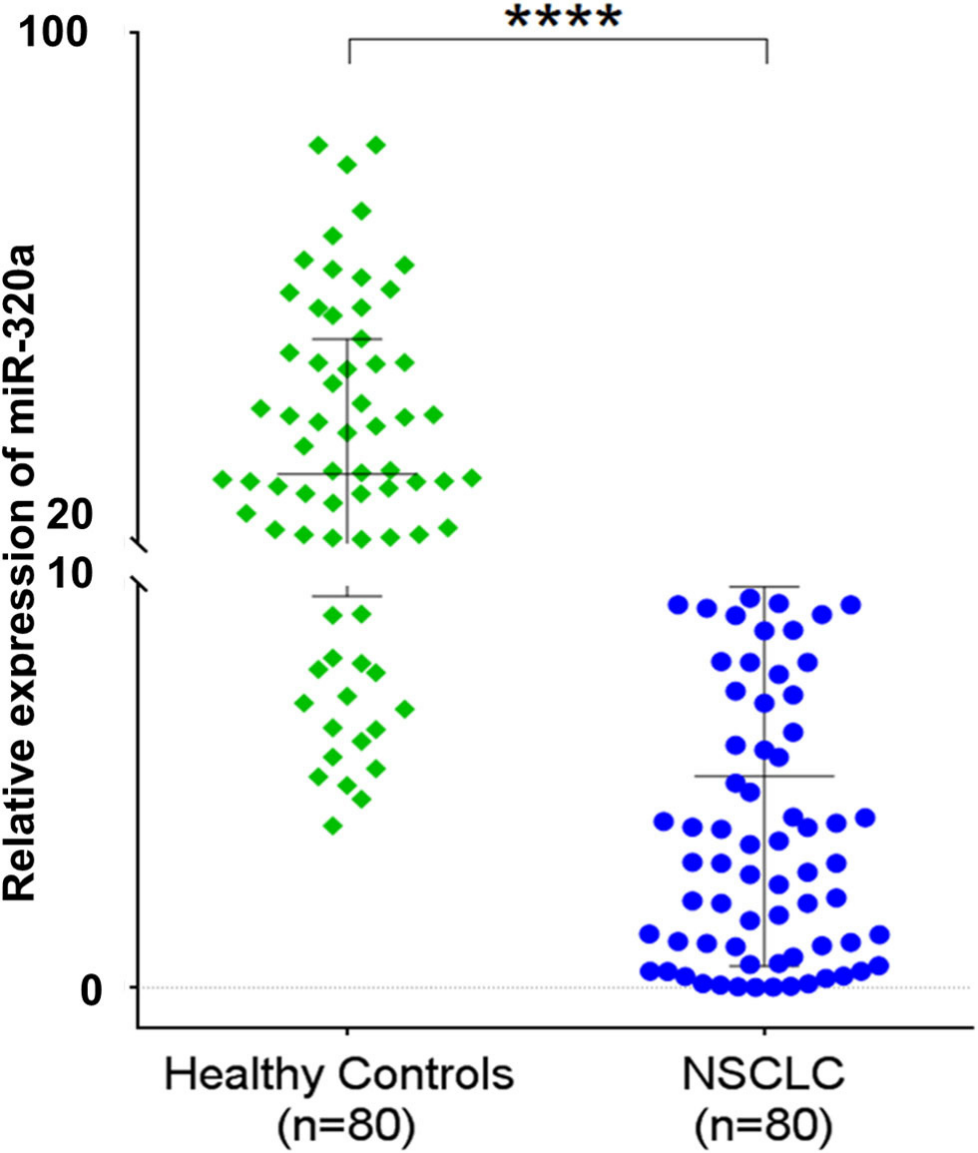
Scatter plot represents a relative expression of circulating miR-320a in plasma samples of NSCLC patients compared to healthy controls. The data are expressed as mean ± SD obtained from at least *n* = 3 independent qRT-PCR experiments. ****p < 0.0001 calculated using an unpaired *t-*test.

miR-320a is transcribed from the gene located at 8p21.3 and is reported to be down-regulated in cancerous tissue compared to adjacent non-cancerous tissue samples of renal (20), glioma (21, 22), breast (23–25), cervix (26), lung (27–29), gastric (30), hepatocellular (31, 32), colorectal (33–35), nasopharyngeal (36) cancers. In this study, our NGS results revealed miR-320a among the top 10 significantly down-regulated miRNAs in NSCLC blood plasma samples compared to the healthy matched control blood plasma samples (data not shown). Further when validated by qRT-PCR, we observed significantly lower levels (~5.87-fold; *p* < 0.0001; 95% CI: −30.38 to −20.82) of circulating miR-320a in NSCLC patients (*n* = 80) compared to the healthy control (*n* = 80) plasma samples (Figure 1). Thus, these results suggested a negative correlation of circulating miR-320a expression with NSCLC.

### Down-Regulated Circulating miR-320a Expression Reflects Poor Prognosis of NSCLC Patients

**Figure 2:**
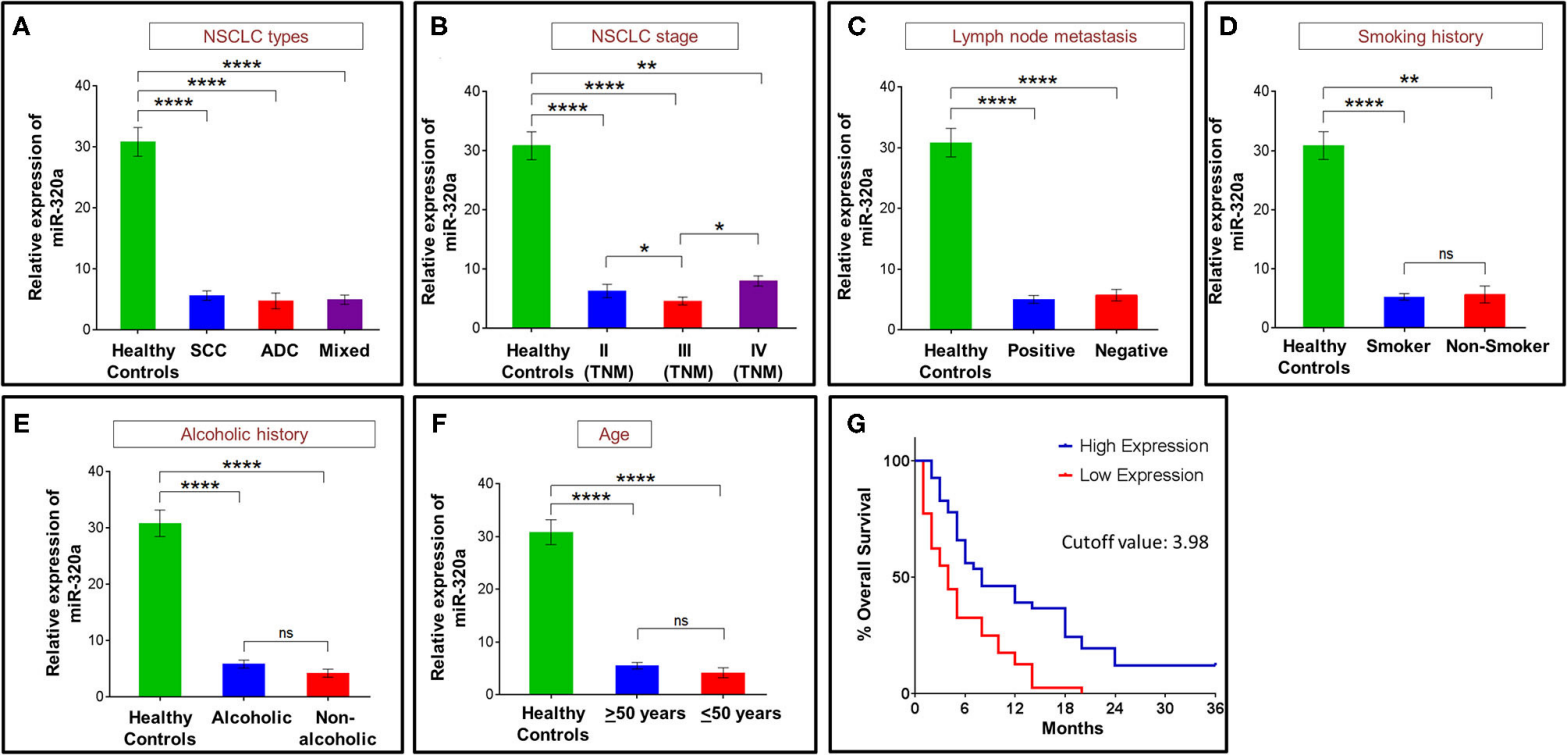
Circulating miR-320a expression correlated with NSCLC patient prognosis. **(A)** NSCLC histological types. **(B)** NSCLC stage. **(C)** Lymph node metastasis status. **(D)** NSCLC patients smoking history. **(E)** Alcoholic history of NSCLC patients. **(F)** Age of NSCLC patients. Bar graphs represent the relative expression of circulating miR-320a from at least *n* = 3 independent experiments. The data are expressed as mean ± SEM where *p < 0.05, **p < 0.01, ***p < 0.001, and ****p < 0.0001 calculated using unpaired and paired *t-*test. **(G)** Kaplan-Meier survival test to determine a correlation between percent overall survival and the circulating miR-320a expression of NSCLC patients. The median expression values of circulating miR-320a in NSCLC patients of 3.98 were used as the cutoff for high and low expression groups [TNM, tumor node metastasis; SCC, squamous cell carcinoma; ADC, adenocarcinoma].

Because miR-320a showed a significant down-regulation in NSCLC patients compared to the healthy controls, we also evaluated any potential associations of its down-regulated levels with NSCLC patients' prognosis. For this, we considered NSCLC subtype, TNM stage, lymph node metastasis and smoking, alcohol and age status of the NSCLC patients. When different NSCLC types were compared, an ~5.5-fold down-regulation was observed in squamous cell carcinoma (*n* = 41; *p* < 0.0001; 95% CI: 18.58–31.85), an ~6.5-fold down-regulation in adenocarcinoma (*n* = 18; *p* < 0.0001; 95% CI: 16.12–36.1), and an ~6-fold down-regulation in the mixed-type (*n* = 21; *p* < 0.0001; 95% CI: 16.71–35.12). In contrast, no significant differences in miR-320a levels were observed between the NSCLC types (Figure 2A). When we evaluated NSCLC stages, patients with TNM stage II cancer showed an ~5-fold down-regulation (*n* = 19; *p* < 0.0001; 95% CI: 14.59–34.54), stage III patients showed an ~7-fold down-regulation (*n* = 54; *p* < 0.0001; 95% CI: 20.47–32.05), and stage IV patients showed an ~4-fold down-regulation (*n* = 7; *p* < 0.005; 95% CI: 6.91–38.83) relative to healthy controls. When compared among stages, only a marginal down-regulation was observed, i.e., between stages II and III (*p* = 0.037), and stages III and IV (*p* = 0.01) (Figure 2B). Similarly, the lymph node metastasis status of NSCLC patients was evaluated where lymph node positive patients showed an ~6-fold down-regulation (*n* = 56; *p* < 0.0001; 95% CI: 20.17–31.54), and lymph node negative patients showed an ~5-fold down-regulation (*n* = 24; *p* < 0.0001; 95% CI: 16.5–33.78) compared to healthy controls. Although the levels of circulating miR-320a in both lymph node positive and negative patients showed significantly lower levels compared to healthy controls, no difference was observed among the two (Figure 2C).

NSCLC patients with a history of smoking showed an ~6-fold down-regulation (*n* = 75; *p* < 0.0001; 95% CI: 20.69–30.56), and non-smokers showed an ~5-fold down-regulation (*n* = 5; *p* = 0.009; 95% CI: 6.331–44.12) of miR-320a when compared to healthy controls. No significant difference was found when both smokers and non-smokers were compared (Figure 2D). Patients with a history of alcohol consumption showed an ~5-fold down-regulation (*n* = 52; *p* < 0.0001; 95% CI: 19.13–30.94), while non-alcoholic patients showed an ~7-fold down-regulation (*n* = 28; *p* < 0.0001; 95% CI: 18.67–34.63) compared to the healthy controls. No significant difference was found among the two groups (Figure 2E). Circulating miR-320a expression levels were also studied to determine correlations with NSCLC patient age. We observed that patients over 50 years showed an ~6-fold down-regulation (*n* = 67; *p* < 0.0001; 95% CI: 20.17–30.6), whereas those below or equal to 50 years showed an ~8-fold down-regulation (*n* = 13; *p* < 0.0001; 95% CI: 15.02–38.43) of miR-320a expression compared to healthy controls (Figure 2F).

In addition to the above prognostic factors, NSCLC patients were grouped into low and high expression groups of circulating miR-320a based on the median cutoff value (3.98). Through Kaplan-Meier and log-rank analyses, the high and low expressing groups were used to evaluate the NSCLC patients' overall survival. The results revealed that patients in the high expression group had a significantly higher median survival compared to the low expression group (*p* < 0.0001; 95% CI: 1.275–3.137) (Figure 2G). Thus, circulating miR-320a expression levels negatively correlated with patient prognosis not only through survival analysis but also through patients' clinico-pathological characteristics such as TMN stages, tumor subtype, lymph node metastasis, and smoking status, suggesting its tumor-suppressive role in NSCLC.

### Effect of miR-320a on A549 Cell Proliferation

**Figure 3:**
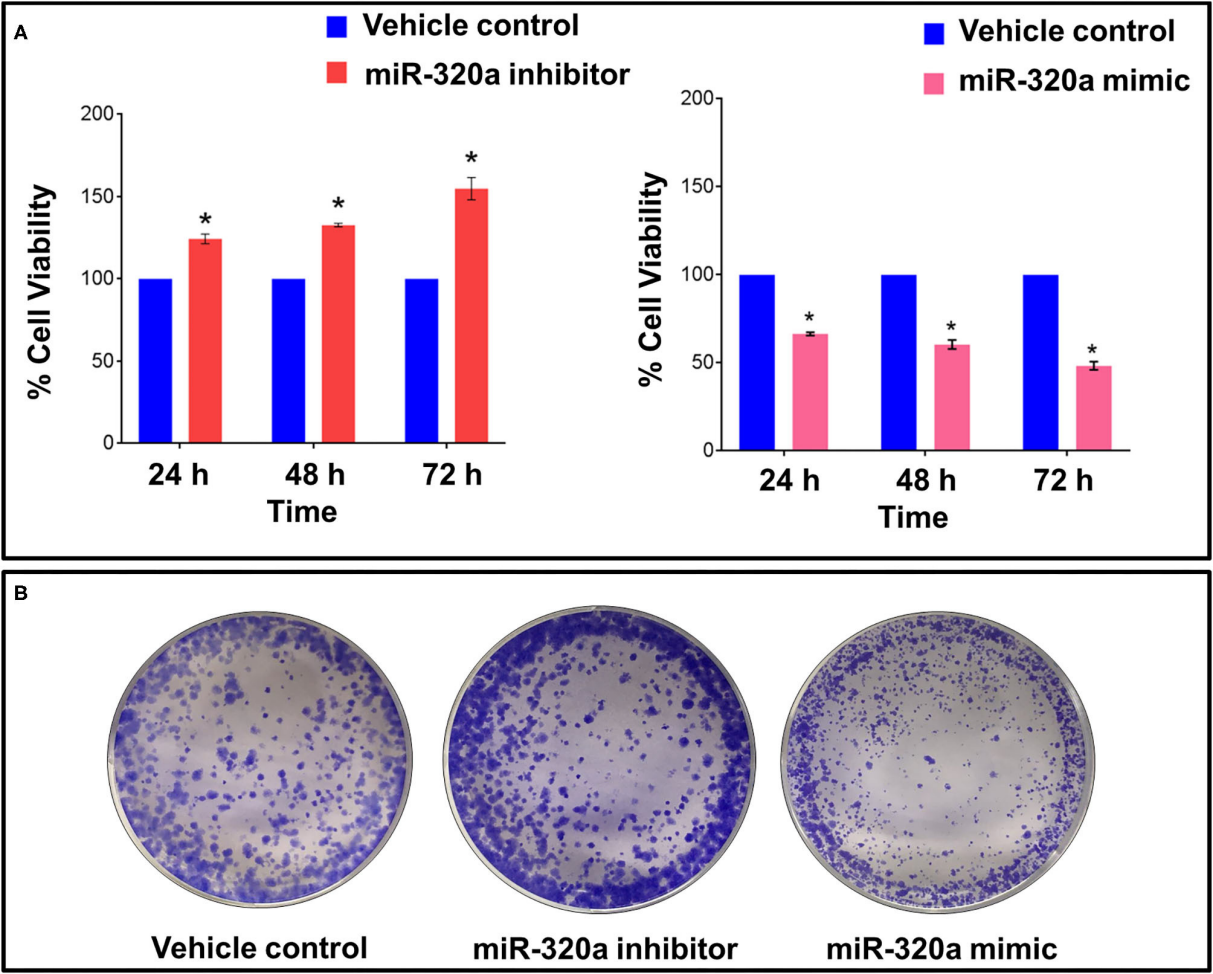
Impact of miR-320a on cell proliferation in A549 cells. A549 cells were transfected with a miR-320a inhibitor (100 nM) or mimic (100 nM) and compared to the vehicle control, as assessed by: **(A)** MTT assays at 24, 48, and 72 h post transfection, expressed as mean ± SEM; and **(B)** representative image of colony forming assays. *p < 0.05.

To determine the effect of miR-320a expression on the proliferation of A549 human lung cancer cells, its levels were suppressed or over-expressed exogenously using an inhibitor or mimic, respectively. The miR-320a inhibitor and mimic were used at concentrations of 1, 5, 10, 20, 50, 100, and 200 nM using Lipofectamine 3000 as a transfection agent, and Lipofectamine 3000 only, as a vehicle control in 96-well plates for 24, 48, and 72 h time points. Ninety six-well plates at each time point were subjected to MTT assays to quantify the proliferation of A549 cells by calculating the absorbance of formazan crystals dissolved in DMSO at 570 nm. We found that the miR-320a inhibitor (at 100 nM) resulted in an ~60% increase in cell proliferation compared to the vehicle control, whereas the mimic (at 100 nM) resulted in an ~50% decrease in A549 cell proliferation compared to the vehicle control at the 72 h time point (Figure 3A). Based on the results obtained, we used 100 nM of the miR-320a inhibitor and mimic and evaluated the cells 72 h post transfection for the experiments discussed below.

Colony forming assays were also evaluated to determine A549 cell growth when transfected with the miR-320a inhibitor (100 nM) or mimic (100 nM). An increase in both the size and number of colonies was observed in cells transfected with the miR-320a inhibitor, whereas the opposite effect was seen when transfected with the mimic compared to the vehicle control (Figure 3B). These assays indicate that miR-320a affects both the growth and proliferation of A549 cells.

### miR-320a Affects Cell Cycle Progression and Apoptosis in A549 Cells

**Figure 4:**
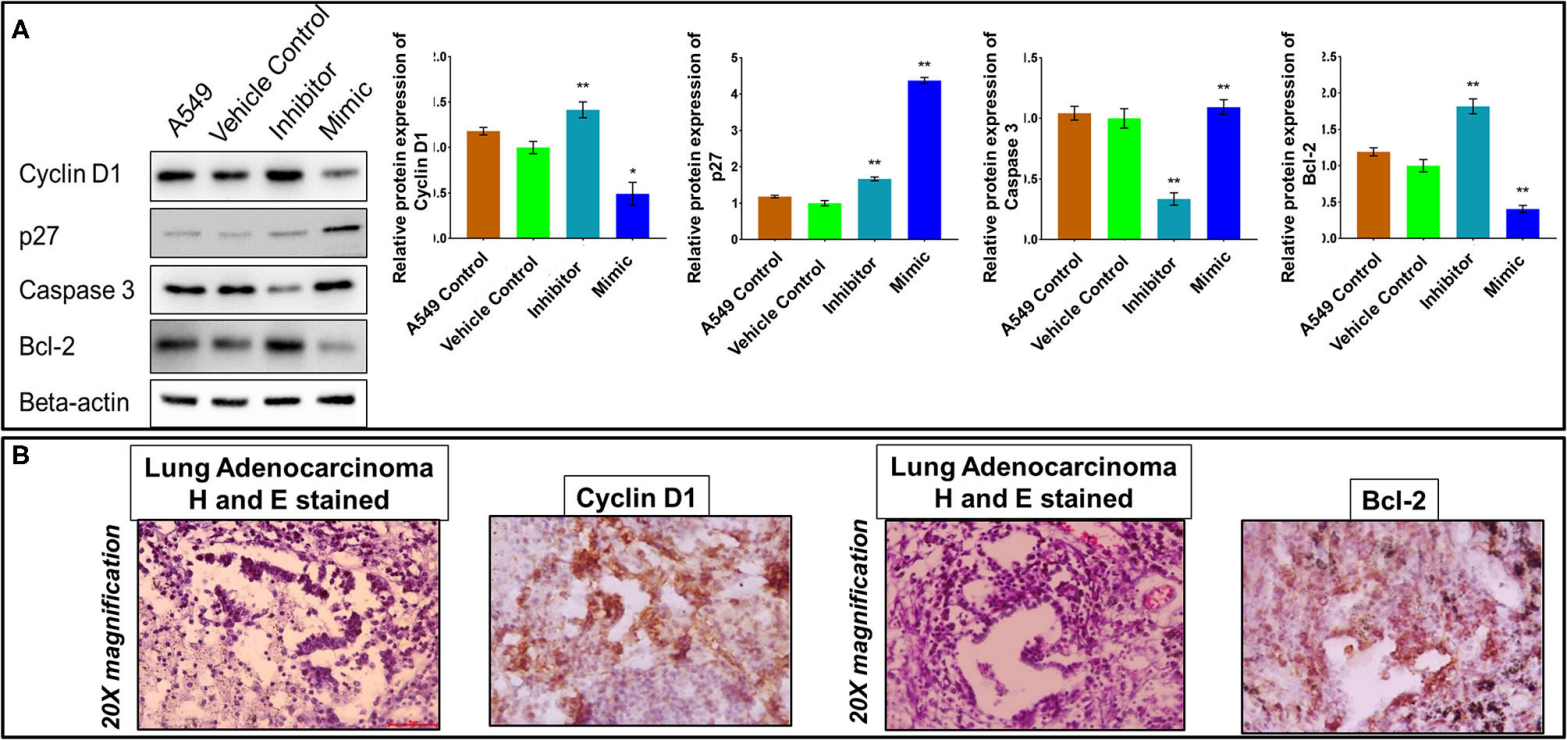
MiR-320a affects cell cycle progression and apoptosis in A549 cells. **(A)** Immunoblot assays (a representative blot is shown) were used to determine normalized protein levels of Cyclin D1, p27, Caspase 3, and Bcl-2 in untransfected A549 cells, A549 cells transfected with vehicle control, a miR-320a inhibitor (100 nM), or a mimic (100 nM). All experiments were carried out three independent times. The data are presented as mean ± SEM where *p > 0.05, **p < 0.05 significant differences from vehicle control calculated using paired *t*-test. **(B)** Immunohistochemistry images represent tumor cells positive (brown-stained) for Cyclin D1 and Bcl-2 proteins in the nucleus and the cytoplasm of NSCLC patient tissue samples. Scale bar 100 μm and *n* = 3 NSCLC tissues.

As assessed through cell proliferation and colony forming assays, miR-320a expression showed an inverse correlation with cancer cell growth and proliferation in A549 cells. We next determined its effects on cell-cycle regulation and apoptosis by evaluating marker proteins in A549 cells. For this, immunoblot assays were performed to measure the levels of Cyclin D1 and p27 for cell cycle analysis in untransfected cells, A549 cells transfected with vehicle control only, and cells transfected with a miR-320a inhibitor (100 nM) or mimic (100 nM). We found that Cyclin D1 levels were increased by ~45% in inhibitor-transfected A549 cells, while it was decreased by an ~50% in mimic-transfected cells. On the other hand, p27 protein levels were higher in A549 cells transfected with the mimic compared to those transfected with the inhibitor, as compared to the vehicle control (Figure 4A). We also performed immunohistochemistry (IHC) to evaluate Cyclin D1 protein levels in NSCLC patient tissue samples, and found that the majority of the tumor cells stained positively for Cyclin D1 (Figure 4B).

The effect of miR-320a on the pro-apoptotic marker, Caspase 3, and the anti-apoptotic marker, Bcl-2 was also evaluated using immunoblot assays. As expected from the previous results, Caspase 3 protein levels were reduced by ~40%, while Bcl-2 levels were increased by ~80% in the inhibitor-transfected A549 cells compared to vehicle control, and vice versa for the mimic-transfected A549 cells (Figure 4A). Moreover, the IHC results of the Bcl-2 protein showed positivity in NSCLC patient tissue samples (Figure 4B). Thus, these results suggested that low levels of miR-320a decreased apoptosis in NSCLC.

### miR-320a Down-Regulation Modulates Multistage NSCLC Tumor Progression

**Figure 5:**
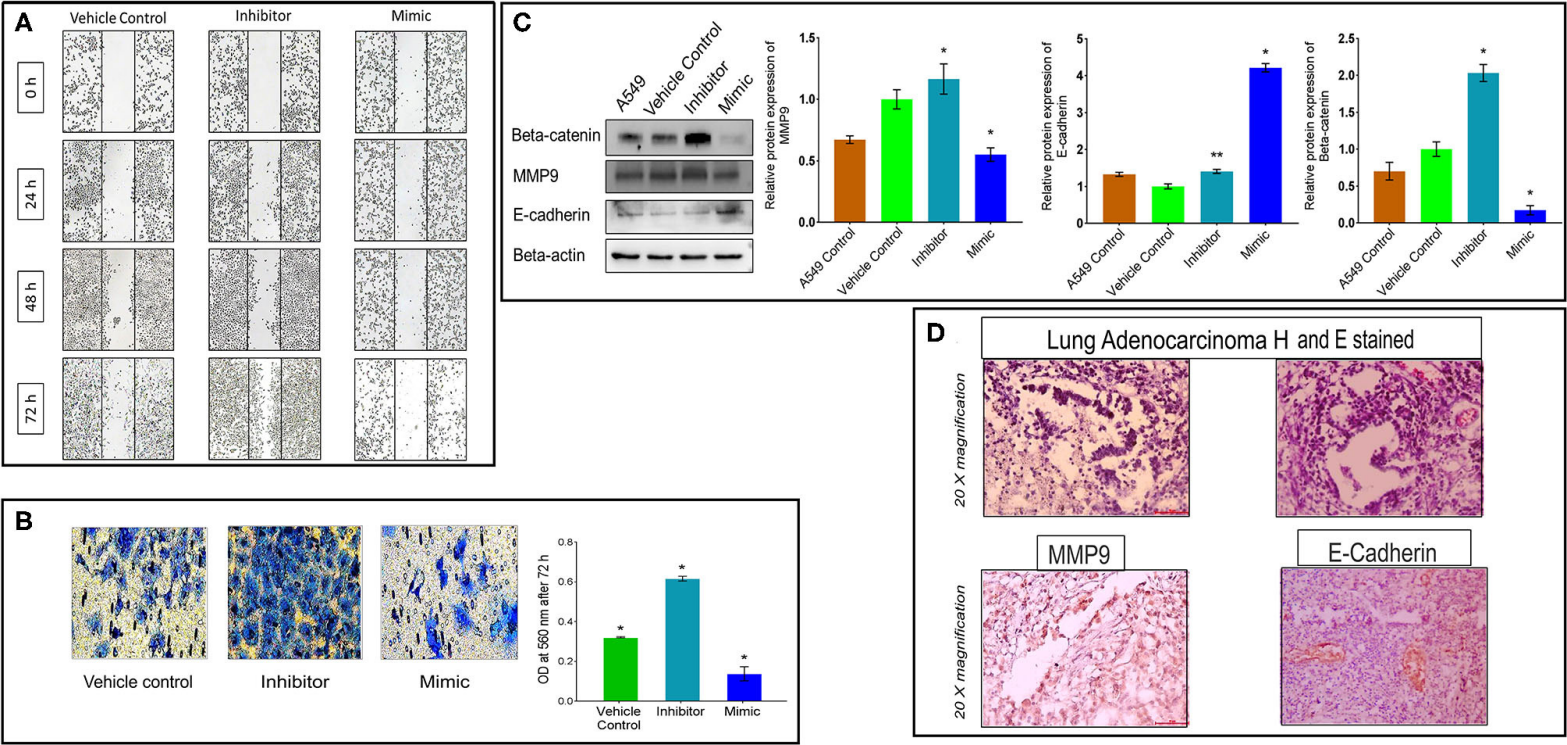
MiR-320a promotes A549 cell migration, invasion, and EMT. **(A)** Cell migration assay in A549 cells transfected with the vehicle control, a miR-320a inhibitor (100 nM), or mimic (100 nM) at 0, 24, 48, and 72 h post transfection. **(B)** Cell invasion assays in A549 cells at 72 h post transfection with the vehicle control, a miR-320a inhibitor (100 nM), or mimic (100 nM). Experiments were performed three independent times. Bar graphs are presented as mean ± SEM where *p > 0.01, **p < 0.001 calculated using paired *t-*test. **(C)** Immunoblot assays (a representative blot is shown) were used to determine normalized protein levels of EMT regulated proteins; Beta-catenin, MMP9, and E-cadherin in untransfected A549 cells, A549 cells transfected with the vehicle control, a miR-320a inhibitor (100 nM), or mimic (100 nM). All experiments were carried out four independent times. The data are presented as mean ± SEM where *p > 0.05, **p < 0.05 significant differences from vehicle control calculated using paired *t*-test. **(D)** Immunohistochemistry images represent tumor cells positive (brown-stained) for MMP9 and E-cadherin proteins in the cytoplasm, and/or the cell membrane of NSCLC patient tissue samples. Scale bar 100 μm and *n* = 3 NSCLC tissues.

To determine the effects of miR-320a on cell tumor progression, we evaluated the migration and invasion potential of A549 cells following transfection with the miR-320a inhibitor or mimic. The results showed an increased cell migration capacity of A549 cells at 72 h post transfection with the miR-320a inhibitor at a concentration of 100 nM, and the opposite effect was observed with the mimic at the same concentration, compared to the vehicle control (Figure 5A). The cell invasion potential was determined by using an assay to measure cell invasion into a matrix and polycarbonate membrane, and the results showed that the miR-320a inhibitor increased the invasion potential of the cells by ~2-fold at 72 h post transfection compared to the vehicle control. In contrast, very few cells evaded the matrix and polycarbonate membrane when the cells were transfected with 100 nM miR-320a mimic (Figure 5B).

Further, we determined the impact of miR-320a on the epithelial-to-mesenchymal transition (EMT), using immunoblot assays and IHC. The results revealed a decreased level of the epithelial marker, E-cadherin, while the level of the mesenchymal marker, Beta-catenin (~100%) and Matrix Metallopeptidase 9 (MMP9) (~20%) increased in the A549 cells in the presence of the miR-320a inhibitor (at 100 nM). Whereas, their levels were significantly reduced in cells transfected with the miR-320a mimic compared to the vehicle control. On the other hand, the E-cadherin levels were significantly increased (~4.2-fold) in the miR-320a mimic-transfected A549 cells compared to the vehicle control transfected cells (Figure 5C). IHC analysis of MMP9 and E-cadherin showed positivity in the tumor cells of NSCLC patient tissue samples (Figure 5D). These results indicated a role of miR-320a in the EMT process, which regulates cell migration and invasion potential in promoting NSCLC progression.

### *AKT3* Is a Direct Target of miR-320a

**Figure 6:**
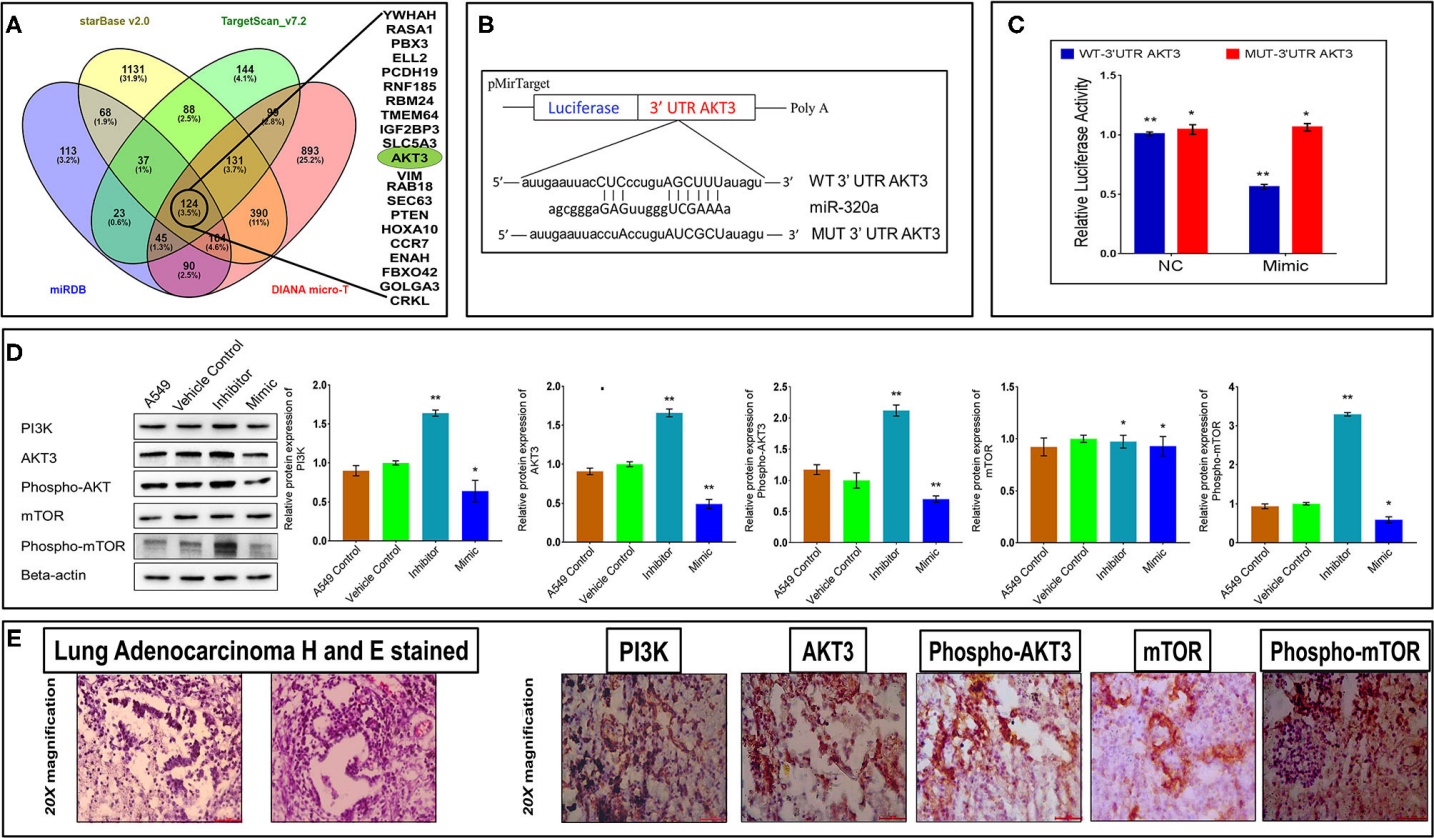
MiR-320a regulates the PI3K/AKT/mTOR pathway through its direct target, *AKT3*. **(A)** MiR-320a *in silico* target prediction shows 124 common targets (inside black circle) common to databases starBase (peach), miRDB (blue), TargetScan version 7.2 (green), and DIANA micro-T (pink). **(B)** MiR-320a binding site with *AKT3* as predicted by DIANA micro-T, an online computational tool. **(C)** Dual-luciferase reporter assays show miR-320a regulating *AKT3* through direct binding. The data are presented as mean ± SEM from three experiments where *p > 0.05, **p < 0.05 significant differences from negative control (NC) calculated using paired *t*-test. **(D)** Immunoblot assays (a representative blot is shown) were used to determine normalized protein levels of PI3K, AKT3, Phospho-AKT3 (pThr305), mTOR, and Phospho-mTOR (pSer2448) proteins in untransfected A549 cells, A549 cells transfected with the vehicle control, a miR-320a inhibitor (100 nM), or mimic (100 nM). All experiments were carried out four independent times. The data are presented as mean ± SEM where *p > 0.05, **p < 0.05 significant differences from vehicle control calculated using paired *t*-test. **(E)** Immunohistochemistry images represent tumor cells positive (brown-stained) for PI3K, AKT3, pAKT3, mTOR, and p-mTOR proteins in the cytoplasm of NSCLC patient tissue samples. Scale bar 100 μm and *n* = 3 NSCLC tissues.

Based on the results from the experiments described above, we concluded that miR-320a acted as a tumor suppressor in NSCLC. To better understand this function, we utilized publicly available databases to predict the oncogenic protein-coding RNA(s) regulating NSCLC tumorigenesis that might be direct targets of miR-320a. TargetScan, DIANA-microT, starBase and miRDB databases predicted 124 common possible targets using Venny 2.1 (http://bioinfogp.cnb.csic.es/tools/venny/) (Figure 6A).

Based on previous reports, *AKT3* is thought to be involved in tumorigenesis of NSCLC (37–43), and from our computational data, we predicted *AKT3* as a direct target of miR-320a binding through its 3′UTR, shown in Figure 6B. To determine if *AKT3* was a target for miR-320a, we used a dual-luciferase reporter assay, and found a significant (~45%) reduction in luciferase activity in A549 cells co-transfected with a miR-320a mimic (100 nM) and pMirTarget WT-3′UTR AKT3 reporter plasmid compared to a miR-320a mimic co-transfected with an empty pMirTarget vector. As expected, the luciferase activity remained unaffected when pMirTarget WT-3′UTR AKT3 plasmid was replaced with pMirTarget MUT-3′UTR AKT3 mutant plasmid (Figure 6C). These results supported the notion that miR-320a directly binds to *AKT3* through its 3′UTR sequence in that *AKT3* RNA levels were inversely correlated with miR-320a levels in the dual-luciferase reporter assays. Additionally, to determine whether this effect was extended to its protein levels and its associated activation in the PI3K/AKT/mTOR pathway, protein levels of PI3K, total AKT3, phosphorylated AKT3 (Thr305), total mTOR, and phosphorylated mTOR (Ser2448) were determined post transfection with the miR-320a inhibitor or mimic in A549 cells through immunoblot assays. It was observed that the levels of both total AKT3 and phosphorylated AKT3 were increased by ~65 and ~120%, respectively, in the inhibitor-transfected cells, whereas their levels significantly decreased by ~50 and an ~20%, respectively, in the mimic-transfected A549 cells compared to the vehicle control 72 h post transfection (Figure 6D). IHC data also showed similar levels of positivity in the cytoplasm of the majority of tumor cells in NSCLC patient tissue samples (Figure 6E). These results suggested that miR-320a not only negatively correlated with *AKT3* mRNA through binding to its 3′UTR region, but also its protein levels.

Further, miR-320a also affected PI3K protein (an upstream molecule to AKT3) levels with an increase of an ~70% and reduction of ~40% in A549 cells transfected with the miR-320a inhibitor or mimic, respectively. When mTOR protein levels were examined (a downstream target of AKT3), only phosphorylated mTOR (Ser2448) showed significant increases and decreases upon transfection with the miR-320a inhibitor and mimic, respectively, compared to the vehicle control, while no change was observed in total mTOR levels (Figure 6D). When NSCLC patient tissue samples were examined for PI3K, total mTOR, and phosphorylated mTOR through IHC, all showed positivity in the majority of the tumor cells (Figure 6E). Thus, these data suggest that miR-320a directly targets *AKT3*, thereby altering the PI3K/AKT/mTOR pathway through a miR-320a/AKT3 axis involved in NSCLC progression and development.

## Discussion

Circulating miRNAs have been found to play essential roles in many biological processes, including cell differentiation, proliferation, apoptosis, and EMT by regulating the expression of various genes associated with cancer development and progression (44). They have also been identified as potential biomarkers that fulfill many recommended properties of successful biomarkers for cancer diagnosis and prognosis (4, 45). However, the mechanistic roles of circulating miRNAs in NSCLC pathogenesis are not yet fully understood. Here, we explored dysregulated circulating miR-320a in NSCLC patient plasma samples and evaluated the underlying molecular mechanisms for their involvement in NSCLC pathogenesis along with their role in prognosis of NSCLC patients. Earlier reports suggested down-regulation of circulating miR-320a in cancers, including NSCLC (~2-fold) (46), colorectal (~3-fold) (47), osteosarcoma (>2-fold) (48), and breast cancer (~2-fold) (49). In contrast, Navarro et al. (50) reported the opposite expression pattern of miR-320a in pancreatic cancer (50). Consistent with the previous reports for NSCLC, colorectal, osteosarcoma cancer (46–48) and breast cancer (49), here we demonstrated that circulating miR-320a levels were significantly decreased (~5.87-fold) in NSCLC patients compared to healthy control blood plasma samples, suggesting its tumor-suppressive role in NSCLC (Figure 1). Circulating miR-320a was also reported to be inversely correlated with cancer stages and osteoblastic subtypes for colorectal and osteosarcoma cancers, respectively (47, 48). Similarly, we found that lower levels of circulating miR-320a were associated with various NSCLC clinicopathological features, including tumor subtypes, TNM stages, and lymph node metastasis. Moreover, its lower expression was associated with poor survival of NSCLC patients (Figure 2G). Additionally, we found that lower levels of circulating miR-320a were associated with various NSCLC clinicopathological features, including tumor subtypes, TNM stages, and lymph node metastasis (Figures 2A–C). These results suggest that circulating miR-320a levels may be predictive of the NSCLC clinical stage, subtype, and lymph node status. Therefore, it may be a suitable candidate for liquid biopsy to replace the highly invasive tissue biopsy for diagnosis/prognosis and treatment response.

Moreover, we observed that patients with age ≤50 years showed significant down-regulation of circulating miR-320a compared to healthy controls (Figures 2E,F). This is in contrast to our recent study on the same patients where we reported that circulating miR-590-5p expression levels in NSCLC patients aged ≤50 years were not significantly down-regulated from healthy controls (11). These discrepancies may be due to the differences in miRNAs types, as different miRNAs regulate the expression of different target genes, and they are also regulated by circular RNA, long non-coding RNA, and miRNAs depending on the specific cellular context (29, 51). Thus, how the age of NSCLC patients might affect the levels of circulating miRNAs in blood samples warrants further investigation.

Here, through the loss- and gain-of-function experiments, we studied the mechanism of miR-320a to regulate NSCLC progression. We demonstrated that the PI3K/AKT/mTOR signaling pathway plays an important role in various cellular functions, such as proliferation, differentiation, and apoptosis. We found that overexpression of miR-320a inhibited cell viability and colony-forming ability of A549 lung adenocarcinoma cells *in vitro* (Figures 3A,B). On the other hand, knockdown of miR-320a in A549 cells effectively increased the levels of Cyclin D1, Bcl-2 (Figure 4A), EMT marker proteins, MMP9 and Beta-catenin, and enhanced cell proliferation, migration, and invasion capacity of the cells. In contrast, a miR-320a mimic resulted in increased levels of Caspase 3 and apoptosis in A549 cells (Figure 4A), accompanied by a reduction in cell migration and an increase in the levels of the epithelial marker, E-cadherin (Figure 5C). Sun et al. (52) showed that miR-320a directly targeted an EMT marker, Beta-catenin and its downstream genes, and was associated with decreased growth of colon cancer cells (49). In addition, miR-320a was shown to be associated with EMT regulation by the suppression of the LIM domain kinase 1 (LIMK1), a serine-threonine protein kinase in lung cancer cells (29, 51). LIMK1 has been shown to participate in the EMT process by affecting the actin cytoskeleton, and was found to be up-regulated in lung cancer cells (29, 51). These data once again suggest that miR-320a acts as a tumor-suppressive miRNA regulating the invasion and migration of lung cancer cells by inhibiting the EMT process. This was also consistent with previous findings that miR-320a acted as a tumor suppressor in breast, colon, and hepatocellular cancer (24, 32, 52).

To date, the functional relevance of miR-320a in lung cancer has not been assessed in detail. Our study demonstrated that miR-320a is involved in the regulation of the PI3K/AKT/mTOR signaling pathway. We observed that *AKT3* was a direct target of miR-320a (Figures 6A–C), and showed that miR-320a suppressed both total AKT3 and phosphorylated AKT3 protein levels in A549 cells (Figure 6D). AKT3 is one of the three isoforms of the AKT family and its overexpression has been reported in breast, prostate, and thyroid cancers (53–55). In the case of breast cancer cells, *AKT3* has been reported as a direct target of miR-29b, where its inhibition attenuated the activation of VEGF and c-Myc proteins (56). However, in NSCLC, it was shown that *AKT3* expression was negatively regulated by miR-217 (42), and thereby led to a decrease in cell proliferation and an increase in apoptosis *via* the PI3K pathway. Similarly, circulating miR-194 negatively regulated phosphorylated AKT protein levels through PI3K and suppressed the PI3K/AKT/FOXO3a pathway, leading to increased cell apoptosis through p53/p21 signaling, resulting in increased levels of Caspase 3/9, p21, and p53 proteins in melanoma cells (57). Recently, it was reported that down-regulation of serum miR-223 led to increased levels of EGFR, which in turn induced the PI3K/AKT pathway in NSCLC patient tissues (58). Zhao et al. (28), also reported that a miR-320a-3p/ELF3 axis regulated the PI3K/AKT pathway and affected cell proliferation, migration, and invasion in NSCLC (28). In addition, miR-320a negatively regulated PI3K/AKT in postmenopausal osteoporosis (59). Similar to these published reports, in this study, we too observed that NSCLC patient tumor tissues were positive for PI3K, AKT3, p-AKT3, mTOR, and p-mTOR proteins by IHC (Figure 6E). Additionally, in miR-320a-inhibitor transfected A549 cells, we observed increased protein levels of PI3K and p-mTOR (Figure 6D). These findings corroborated that the miR-320a/AKT3 axis modulated the PI3K/AKT/mTOR pathway in NSCLC impacting both tumorigenesis and tumor progression.

**Figure 7:**
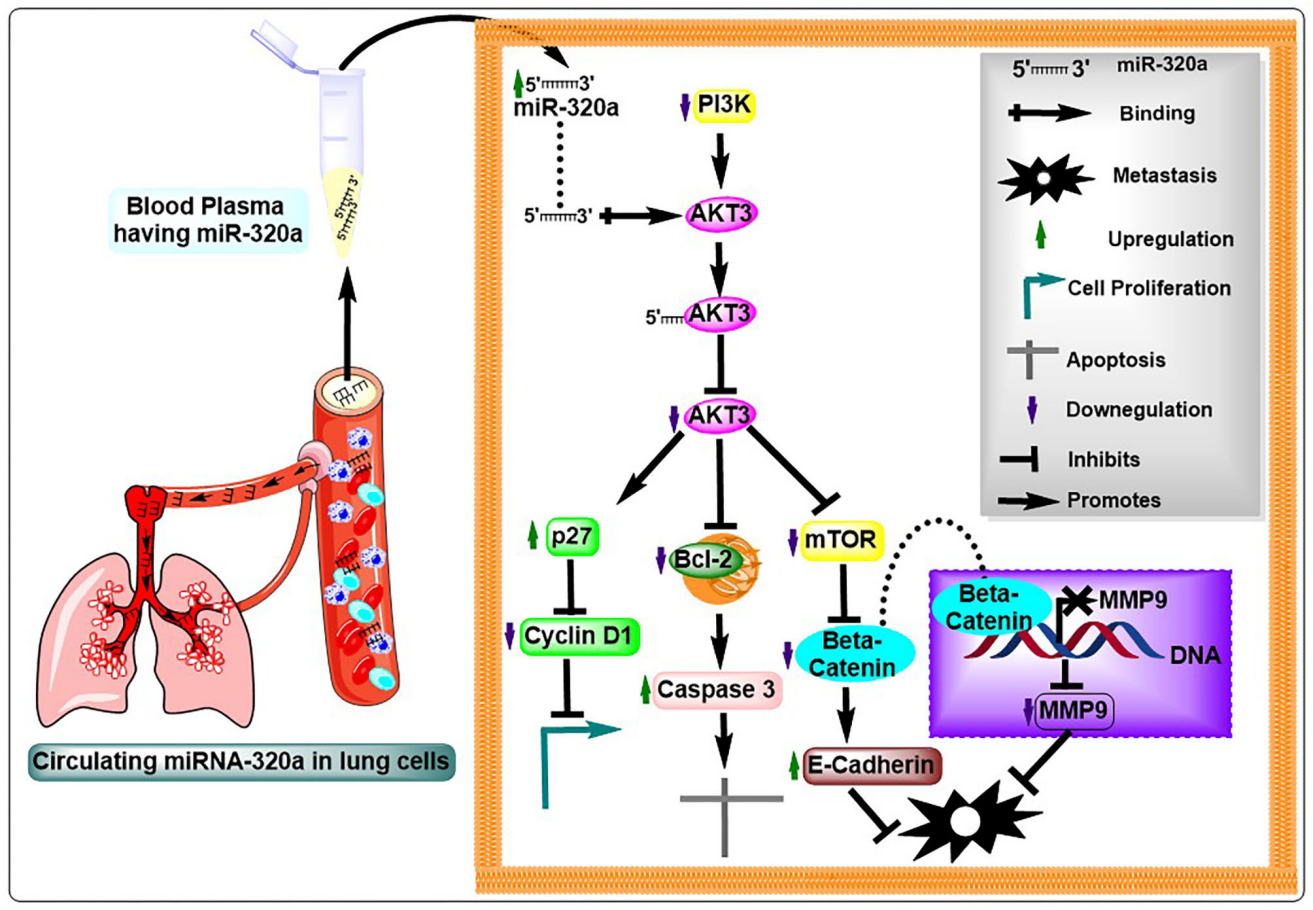
Illustration of the potential mechanistic roles of miR-320a in NSCLC pathogenesis. Circulating miR-320a binding to the 3′UTR of *AKT3* results in an increase or decrease in its downstream molecules represented by up and down arrows, respectively, thereby, influencing the AKT3-associated molecules to impact hallmarks of cancer in NSCLC tumorigenesis.

In conclusion, our findings demonstrated that circulating miR-320a acts as a tumor suppressor in NSCLC and may be used as a prognostic factor in these patients (Figure 7).

## Data Availability Statement

The raw data supporting the conclusions of this article will be made available by the authors, without undue reservation.

## Ethics Statement

The present study was approved by the Ethics Committee of the Regional Cancer Centre, Indira Gandhi Medical College, Shimla, Himachal Pradesh, India, and the Central University of Punjab, Bathinda, India. The patients/participants provided their written informed consent to participate in this study.

## Author Contributions

AJ and AK conceived the original idea and planned the experiments. AK carried out the experiments and wrote the manuscript with support from AJ. US prepared conclusion figure and helped in formatting manuscript and its figures. TB formatted the references. RS and MG provided the clinical samples and their data. MR provided expertise in immunohistochemistry experiments. AJ and KV provided critical feedback. AK, AJ, US, TB, and KV contributed to the final version of the manuscript. AJ supervised and supported the research. All authors contributed to the article and approved the submitted version.

## Conflict of Interest

The authors declare that the research was conducted in the absence of any commercial or financial relationships that could be construed as a potential conflict of interest.
